# Comparative Analysis of Elastic Polyester Sportswear Fabrics with Printed Graphene Patterns

**DOI:** 10.3390/ma16052028

**Published:** 2023-03-01

**Authors:** Simona Vasile, Magdalena Georgievska, Cosmin Copot, Alexandra De Raeve

**Affiliations:** 1 Fashion and Textiles Innovation Lab (FTILab), HOGENT University of Applied Science and Arts, 9051 Ghent, Belgium; 2 Department of Materials, Textiles and Chemical Engineering, Center for Textile Science and Engineering, Ghent University, 9052 Ghent, Belgium

**Keywords:** graphene-printed patterns, sportswear fabrics, thermophysiological comfort, sensorial comfort

## Abstract

In this study two elastic polyester fabrics differentiated by a graphene-printed pattern, called honeycomb (HC) and spider web (SW), were analyzed with a focus on their thermal, mechanical, moisture management and sensorial properties, aiming to identify the fabric with the most elevated heat dissipation and comfort for sportswear. The shape of the graphene-printed circuit did not lead to significant difference between the mechanical properties of the fabrics SW and HC assessed by the Fabric Touch Tester (FTT). Fabric SW outperformed fabric HC with respect of drying time, air permeability, moisture, and liquid management properties. On the other hand, both the Infrared (IR) thermography and FTT-predicted warmness clearly showed that fabric HC dissipates heat faster on its surface along the graphene circuit. This fabric was also predicted by the FTT as smoother and softer than fabric SW and had a better overall fabric hand. The results revealed that both graphene patterns resulted in comfortable fabrics with great potential applications in sportswear fields, in specific use scenario’s.

## 1. Introduction

Many textile products, from beddings to sportswear, need a cooling function to be able to rapidly dissipate heat and sweat from the body through the different pathways, such as conduction, convection, evaporation, and radiation [1]. Conductive heat transfer in textiles happens through direct contact between solid surfaces. It could have a significant contribution to the total cooling, especially when there is a greater temperature difference between the body and environment or surfaces in direct contact [2]. In IR-opaque textiles, heat conduction surpasses radiative heat transport and can give effective cooling in ambient temperatures from 18 °C to 36 °C [1]. Most conventional textiles have very poor thermal conductivity [3]. In sportswear, a variety of natural and synthetic fibers are conventionally used with low thermal conductivities of around 0.033 to 0.10 W m${}^{-1}$ K${}^{-1}$ [1,4]. Thermal conductivities of natural hemp, viscose, linen, cotton and bamboo fabrics are reported to be around 0.022, 0.031, 0.043, 0.026–0.065, and 0.039–0.045 W m${}^{-1}$ K${}^{-1}$, respectively [1,5,6,7]. The thermal conductivity can be increased by minimizing the trapped air by dense and thinner structures, increasing polymer chain alignment, or by introducing conductive fillers [1].

Graphene (G) has become popular for textile applications due to its multifunctionality and remarkable properties including high thermal conductivity (above 3000 W m${}^{-1}$ K${}^{-1}$), mechanical strength, antistatic effect, UV-protection, electrical conductivity and easy functionalization. It consists of 2D single carbon atoms arranged in a lattice honeycomb structure with single bond length of 0.142 nm [8]. Graphene is attractive for lightweight sportswear or personal protective clothing applications, due to its multifunctionality. Thus, it has been used as a conductive filler for increasing heat transport in textiles [9,10]. Molina [11] summarized all the work performed in the area of G fabrics, focusing on the two most widely employed production techniques: chemical deposition (coating) and chemical vapor deposition (CVD) of G, GO (graphene oxide), or rGO (reduced graphene oxide) on fabrics. GO shows much better thermal conductivity when it is reduced than in the unreduced state [3]. Coating is a simple, commonly applied, scalable method for applying graphene on conventional fabrics, allowing them to remain flexible and comfortable. Dip-coating applies the filler on both sides of the fabric, while methods functionalizing one side of the fabric could be more suitable for preserving the flexibility and breathability. In any case, it is important that the conductive filler binds strongly to the fibers to prevent leakage [4,12]. Abbas et al. [4] compared cotton fabrics coated with G, MWCN (multi-wall carbon nanotubes) and boron nitride (BN) fine particles, concluding that G showed the best improvement of the thermal conductivity (by 2.6 °C), not influencing the hydrophilicity of cotton. Adding GO in various formulations has led to an increased thermal conductivity of polyamide, polyester/cotton, merino wool/nylon and thiol-modified cotton fabrics to 0.521, 0.7, 0.81, and 2.13 W m${}^{-1}$ K${}^{-1}$, respectively [13,14,15,16]. Eco-friendly fabrics from plain weave bamboo viscose, coated with graphene/cellulose nanocrystals were indicated as suitable for rapid cooling, with thermal conductivity of 0.136 W m${}^{-1}$ K${}^{-1}$ [1,12]. A graphene-based conductive layer (20 wt% GNP and 20 wt% BN) coated on the inside of a T-Shirt, covered with an active cooling source increased the spreading and transfer of the cool air to the skin [11]. In addition to improving thermal conduction, multilayer graphene (MLG) has been also used as a conductive layer for camouflage and adaptable thermal radiation in textiles [17].

Screen printing of G has been employed mainly to create sensors and electrodes for smart textiles [18,19,20]. For instance, a water-based ink with graphene was deposited on a knitted polyester-elastane fabric for smart textile strain sensors [18] and on cotton fabric for ECG dry electrodes, respectively [19]. Li et al. [20] deposed graphene ink via airflow assisted screen printing on spandex/cotton fabrics for electroconductive textiles with better flexibility and air permeability, washing durability and stability. In a very recent study Wei et al. [21] reported improvements of the thermal protection performance of graphene screen printed cotton fabric for welding protective clothing. There are some toxicity concerns with graphene based materials, however they are found in vitro applications, such as cancer treatment, drug delivery, gene transport, and cell culture, without significant toxicity [9].

Commercial graphene-based textile fabrics and clothing are emerging. A few examples include: the patented planar thermal circuit invention (G+^®^ Planar Thermal Circuit^®^) by Directa Plus [22], the G+ cycling jersey by Oakley and Bioracer, Alè’s R-EV1 Velocity cycling garments, products by Colmar (sky jackets), Ryzon’s triathlon Verge Aero Sleeve Tri Suit and Sonar Graphene Swimsuit, EE sports patented 3LAYEER^®^ fabric, Alfredo Grassi’s workwear and Romy Calzado’s printed denim collection. Sportswear printed with graphene-based inks by BiaBrazil are expected to be launched in 2023, in agreement with UK-based developer of inks, Versarien, and are claimed to increase the moisture management properties and thermal transmittance by 18% [23].

While recent studies [18,19,20,24] reported about screen printing graphene on various substrates for the purpose of electrical conductive smart textile sensors and electrodes, the number of studies dealing with the thermal properties of graphene fabrics is very limited. Presently, graphene is emerging in some commercial sportswear and claims are made about their performance and comfort. Nevertheless, there is a lack of studies about that and this is limited to dip-coated cotton fabrics [4]. In addition to good thermal management, moisture and air management, as well as fabric hand, are tremendously important for sportswear. Therefore, in this study two polyester-elastane (PES-EL) woven fabrics differentiated by their graphene-printed pattern were investigated focusing on their physical, thermal and comfort properties, for passive thermoregulation. Differences in fabric properties attributed to the graphene patterns were highlighted aiming to select the most comfortable fabric with high dissipation and moisture management properties for sportswear.

## 2. Materials and Methods

### 2.1. Materials

**Figure 1:**
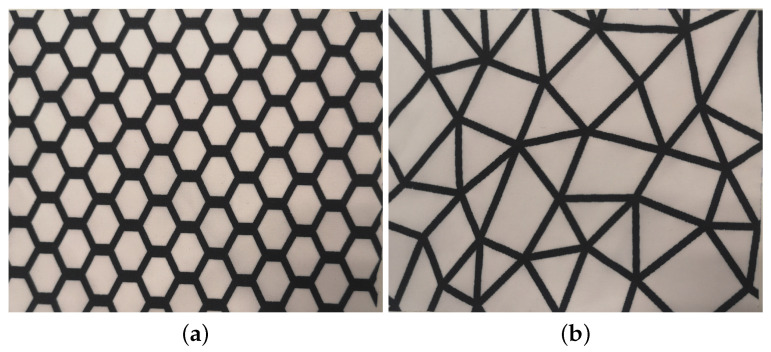
Digital images with a resolution of 2870 × 2680 for 10 cm × 10 cm fabric (**a**) honeycomb (HC) and (**b**) spiderweb (SW).

**Figure 2:**
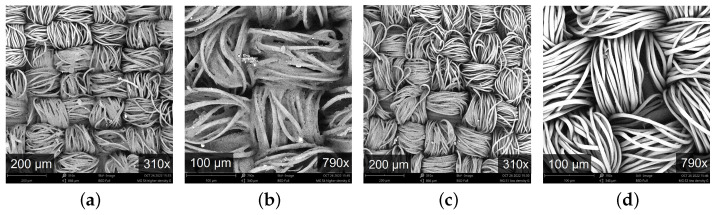
SEM images of PES-EL fabric with (**a**) honeycomb (HC) graphene pattern with resolution ×310; (**b**) honeycomb (HC) graphene pattern with resolution ×790; (**c**) spiderweb (SW) graphene pattern with resolution ×310 and (**d**) spiderweb (SW) graphene pattern with resolution ×790.

Two PES-EL woven fabrics with a plain weave, differentiated by their graphene-printed patterns, were considered in this study. According to the technical information from the textile manufacturer, a prepared rOxide graphene was purchased and printed directly on the fabric substrate, using two patterns hereafter called honeycomb (HC) and spiderweb (SW), as shown in Figure 1. Structural and physical parameters of the two fabrics are shown in Table 1 and were assessed as further described in Section 2.2.1. Figure 2 shows SEM images of the PES-EL fabrics with a HC graphene pattern (a), (b), and SW graphene pattern (c) and (d).

### 2.2. Methods

#### 2.2.1. Fabric Physical Properties

The physical properties of the fabrics used in this study were assessed as described below:

- The graphene content was calculated using basic image processing techniques. In the first step, image acquisition was performed for both fabrics on an equal surface of 1 $m{}^{2}$. Next, using Matlab^®^ software, the acquired images were converted to black and white images from which the graphene content was calculated using the following equation:
  (1) $$Graphene\_content=\frac{no\_of\_black\_pixels\times 100}{no\_of\_total\_pixels}$$
- Fabric mass per unit area was assessed according to ISO 3801-1977. In total, 10 square specimens (10 × 10 cm) were measured with an accuracy of 0.001 g. The mean of 10 measurements was expressed to the nearest gram.
- Thickness of the fabrics was assessed according to ISO 5084-1996 under a pressure of 1 kPa using a Hess thickness gauge model HDM-3 with an accuracy of 0.01 mm. The mean value of 10 measurements was expressed to the nearest 0.1 mm.
- Bulk density of the fabrics (kg·m${}^{-3}$) was calculated as ratio of fabric mass per unit area (g·m${}^{-2}$) and thickness (mm).
- Fabric elongation was evaluated according to EN 14704-1-2005 method A, using rectangular fabric strips (50 × 300 mm) and an Instron tensile tester with the gauge length of 200 mm. The extension and retraction rate of the specimen was set at 100 mm/min and the required cycling limits between gauge length and a load of 30 N (corresponding to 6 N/cm width). The fabric elongation in weft and warp direction was calculated for the fifth cycle. The mean value of five measurements was expressed to the nearest 0.1%.

#### 2.2.2. Fabric Mechanical Properties

Several mechanical, surface, and thermal properties of the two fabrics were assessed using the Fabric Touch Tester (FTT) equipment. FTT is an integrated instrument that simultaneously measures four kinds of fabric properties of the fabric namely compression, bending, surface, and thermal properties, expressed by 12 fabric indices, as shown in Table 2. Details about the measuring modules of the instrument and calculation of the indices are given elsewhere [25,26].

Fabric indices related to bending, surface friction and roughness are simultaneously measured in weft (e) and warp (a) direction. All fabric indices are measured for the inside and outside of the fabric. In our case, the outside of the fabric refers to the graphene-printed side. To benefit the thermal properties of the graphene-pattern and distribute the excess of body heat, this side should be in close contact with the body. The measured fabric indexes are normalized (value between 0 and 1) by FTT software and classified into five grades: 1—very low, 2—low, 3—middle, 4—high, and 5—very high) [27]. In total 10 specimens (L-shape form, 31 × 31 cm) were prepared and tested, 5 for each side of the fabrics. No testing standard currently exists for the FTT, therefore the fabrics were tested according to the testing protocol of the equipment manufacturer.

#### 2.2.3. Fabric Thermal Properties

The FTT device also has a thermal module consisting of two plates in between which the fabric is placed. A heater is installed in the upper plate and when a 10 °C temperature difference is achieved between the upper and lower plates, the upper plate will move downwards to compress the testing specimen and then return upwards (recovery). The FTT device measures the maximum thermal flux Qmax (W/m${}^{2}$) during the compression phase (about half of the total 120 s testing period). Qmax value represents the sensation of coldness or warmth felt when skin is touching an object. Thermal conductivity (W/m °C) of the specimens is calculated both during the fabric compression (TCC) and fabric recovery (TCR) phase.

**Figure 3:**
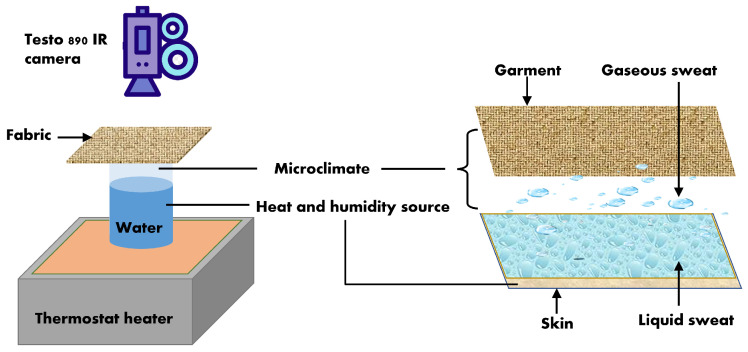
Instrumental setup to assess heat dissipation along the graphene circuit using IR thermography.

Moreover, the thermal properties of the two fabrics were investigated by IR thermography. Thanks to its elevated thermal conductivity, when applied in contact with the body, the graphene thermal circuit provides dissipation of the heat excess and heat equalization. The changes of fabric surface temperature enabled by the graphene-circuit were recorded by an IR thermal camera Testo, model 890-2 with a thermal sensibility <40 mK at +30 °C [28]. A test setup was used similar to reported in literature [13]. The sample was placed on a recipient filled with water at 35 °C temperature, simulating human skin temperature, with the graphene pattern facing the heated water without touching it. The camera was positioned on a level tripod, perpendicular to the region of interest, at a distance of about 25 cm. Thermograms and video’s were recorded and analyzed offline by the Testo software IRSoft version 4.5. The emissivity was set at 0.95. The heat dissipation along the HC and SW-graphene circuit was monitored during one minute exposure to heat. To assess the fastest heat distribution along the two graphene circuits, the temperature profile along a specified horizontal line across the IR thermograms was computed for both fabrics and the temperature gradient at certain time intervals was calculated. The instrumental setup used to assess the heat dissipation along the graphene circuit of the fabrics is illustrated in Figure 3.

#### 2.2.4. Fabric Thermophysiological and Sensorial Comfort Properties

- Air permeability in relaxed and stretched fabric state

Fabric air permeability was determined as velocity (mm/s) of an air flow passing perpendicularly through a test specimen under specified conditions (ISO 9237-1995). Air permeability of the two fabrics was measured at an air pressure drop of 100 N on a fabric test surface of 20 cm${}^{2}$ using an air permeability tester (EMI Development). The studied fabrics contain a large amount of elastane to enable stretching during use. Air permeability was therefore measured both on the fabrics in relaxed state (RS), as well as exposed to bilateral stretch of 10% (BS10) and 20% (BS20). To apply biaxial stretch, a device was used as reported elsewhere [29]. Five specimens were tested for each state (RS, BS10 and BS20) and the mean air permeability was calculated.

- Water vapor permeability

Water vapor permeability WVP (g m${}^{-2}$ Pa${}^{-1}$ h${}^{-1}$) was determined by inverted cup method, according to ISO 15496-2018. A fabric specimen (180 mm diameter) was fitted together with a waterproof, highly water-vapor permeable, hydrophobic membrane on a ring holder and then placed in a water bath for 15 min, with the membrane touching the water. The side with the graphene pattern was oriented towards the bath. A cup containing a saturated potassium acetate solution, creating a relative humidity of 23% at the specimen’s upper face and covered with a second piece of the same membrane, was weighed and then inverted above the specimen in the ring holder, so that the membrane was in contact with the specimen. A transfer of water vapor through the specimen from the water side to the cup took place. After 15 min the cup was taken off and re-weighed. For each fabric quality SW and HC three specimens were tested and the mean value of WVP calculated.

- Moisture drying time

This was determined according to ISO 17617-2014, vertical drying method A2 where the wet specimen is exposed to the test environment (20 °C, RH 65%, air velocity less than 0.1 m/s) from both sides. Three square test specimens (100 × 100 mm) were prepared for each fabric quality and conditioned for minimum 24 h in the standard atmosphere at 20 °C and RH 65%. Two perspiration solutions, i.e., alkaline ALP (pH 8 ± 0.2) and acid ACP (pH 5.5 ± 0.2) were prepared according to ISO 105-E04: 2013 and conditioned for 24 h at 20 ± 2 °C. The dry weight of the sample was recorded and then a predefined quantity of artificial perspiration was applied in the center of the exposed upper surface (i.e., graphene side). The weight of the wet sample is measured at a 5 min interval, for a total of 60 min test period. Drying time is the time (min) for which 100% of the water loss occurs. For each perspiration solution (ALP, ACP) three specimens were tested and the mean was calculated.

- Moisture management properties

Moisture management properties (AATCC 195-2011) of the fabrics were assessed by a Moisture Management Tester (MMT). A fabric specimen is placed between two horizontal (upper and lower) electrical sensors each with seven concentric pins. A predetermined amount of conductive test solution that aids the measurement of electrical conductivity changes is dropped onto the center of the upward-facing test specimen surface (graphene side). During 120 s, the test solution is free to move in three directions: radial spreading on the top surface, movement through the specimen from top surface to the bottom surface, and radial spreading on the bottom surface of the specimen. During the test, changes in electrical resistance of the specimen are measured and recorded. The MMT gives a comprehensive profile of a fabric’s liquid moisture management capability by measuring several indices, such as wetting time (WT), maximum wetted radius (MWR), spreading speed (SS), absorption rate (AR) on the top (inner) and bottom (outer) surface of the fabrics and by calculating the accumulative one-way transport capability (R) and Overall Moisture Management Capacity (OMMC). A grading 1–5 (low–high) is applied to all these indices. Based on these parameters MMT distinguishes seven categories of fabrics from waterproof fabric to moisture management fabric. The 10 specimens of 8 cm × 8 cm were tested and the mean value and STDEV were calculated for all parameters.

- Sensorial comfort indices

The fabric indices measured by FTT instrument (Table 2) are subsequently used by the FTT software to predict three primary comfort indices (i.e., smoothness, softness, and warmth) and two global comfort indices (i.e., total hand and total feel). Primary comfort indices are computed separately for active (hand) and passive (touch) evaluation in which results related to fabric outside (O) and, respectively, inside (I) are considered. Passive and active evaluation means wearing the fabric on the skin or assessment of the fabric with the hand, respectively. The total hand and total touch are calculated by the FTT based on the three primary comfort indices corresponding to active and passive evaluation, respectively. Details about the calculation of these comfort indices are given elsewhere [25,26]. The primary and global comfort indexes are normalized (value between 0 and 1) and then classified into five grades 1—very low, 2—low, 3—middle, 4—high, and 5—very high) [27]. In total 10 specimens were tested, 5 for each side of the fabrics.

All specimens were conditioned prior to testing for a period of 24 h, at 20 ± 2 °C and 65 ± 4% relative humidity (ISO 139:2005) in a conditioning chamber. A paired *t*-test was used to identify statistically significant differences (α = 0.05) between the fabric properties due to the graphene pattern (*p*-value > 0.05 indicates no statistical significant difference).

## 3. Results and Discussions

In this section, the results of the tests performed according to the methods in Section 2.2 are presented and discussed for the fabric physical Section 3.1, mechanical Section 3.2, thermal Section 3.3, and comfort Section 3.4 properties.

### 3.1. Fabric Physical Properties

The measured physical and structural properties of the two fabrics are presented in Table 1, Section 2.1. The two graphene patterns were applied on the same substrate 74/26 PES/ EL woven fabric with a plain weave, which can be seen in the SEM images in Figure 2. The more dense HC-graphene pattern (i.e., 30.5%·m${}^{-2}$ versus 23.9%·m${}^{-2}$) leads to a significant (*p* = 0.00) higher mass per unit area (120 g·m${}^{-2}$) as compared with fabric SW (112 g·m${}^{-2}$). The small differences in fabric thickness were also statistically significant (*p* = 0.00). Fabric HC exhibited a higher bulk density (382 kg·m${}^{-3}$) than fabric SW (378 kg·m ${}^{-3}$) but this difference was not statistically significant (*p* = 0.23). The elongation of the two fabrics was significantly different, especially in warp direction. The graphene pattern HC causes more differences (direction wise) in fabric’s elongation than the graphene pattern SW.

### 3.2. Fabric Mechanical Properties

Mechanical and surface properties derived from the FTT fabric indexes are reported in Table 3. Fabric bending (BAR, BW), friction (SFC) and roughness (SRA, SRW) indexes are reported in weft (e) and warp (a) direction. Compression (CW, CRR, CAR, and RAR) and thermal (TCC, TCR, and Qmax) indexes are not dependent on the fabric direction. All fabric properties are referring to the fabric outside with the graphene-printed pattern and fabric inside.

For the fabric outside with the graphene pattern, a paired *t*-test (α = 0.05) showed no statistically significant differences between most of the indexes of the two fabrics (Table 4), except for bending work in warp direction BW${}_{a}$ (*p* = 0.002). Despite the same substrate, it seems that significantly higher bending work is needed to bend HC-fabric as compared with SW-fabric and that could be due to the more dense graphene pattern. Nevertheless, despite these differences, both fabrics were classified as having very low bending work BW (grade 1) by the FTT software (Table 5). The fabric direction had a significant influence only on the bending properties (BAR) of the fabric SW. A higher force is needed to bend this fabric in the warp direction. This pattern is less uniform than pattern HC and that could explain these direction-based differences.

There were no significant differences between the surface properties (friction and roughness) of the two fabrics for the outside. The friction coefficient SFC of the fabric HC was significantly different (*p* = 0.01) depending on direction. The FTT classified both fabrics as having low surface friction (Table 5) regardless of direction. Large variance of compression properties was noticed (Table 3), especially for SW-fabric but the differences between the compression indexes of the two fabrics were not statistically significant.

For the fabric inside, the bending properties of the two samples were not significantly different (Table 6), except the fabric bending work in the weft direction BW${}_{e}$ (*p* = 0.008). As it can be seen in Table 5, fabric HC was categorized as having a low BW${}_{e}$ (grade 2) while fabric SW had a very low BW (grade 1). The more dense graphene pattern applied to the outside of the fabric HC seems to lead to different bending properties when the fabric is bent along the printed or unprinted side. The fabric direction seems to also significantly influence the bending work BW (*p* = 0.033) of this fabric HC (Table 6).

There were no significant differences between the surface properties (friction and roughness) of the two fabrics, except for the surface roughness amplitude in the weft direction SRA${}_{e}$ (*p* = 0.002). Fabric SW performed better than fabric HC and was categorized as having very low roughness (grade 1) regardless of direction. The friction properties of both fabrics (SFC) at the inside were significantly different depending on the fabric direction (Table 6).

Based on the FTT-grading, it can be concluded that both graphene-printed fabrics have comparable (very low to low) bending, friction, and roughness properties.

### 3.3. Fabric Thermal Properties

#### 3.3.1. FTT Measurements

The FTT equipment compresses the fabric between the two plates with a temperature gradient of 10 °C (upper plate is 10 °C warmer than lower plate). The heat flux passes transversally through the fabric during the compression phase which lasts about 60 s. The maximum heat flux Qmax recorded is used to calculate TCC and TCR. These had a value between 0.032 W/m °C and 0.034 W/m °C (Table 3). The thermal properties of the fabrics were significantly different only at the fabric inside TCC (*p* = 0.023) and TCR (*p* = 0.008). Both fabrics were classified as having low thermal conductivity (grade 2), regardless of the fabric side (Table 5).

Abbas et al. [4] reported higher values of 0.078 W/m.K for cotton woven fabrics coated with graphene, but the fabrics in their study were coated with graphene on the whole surface, the thermal conductivity was estimated and a test setup with a different principle was used. Lu et al. [30] reported similar values to our graphene-functionalized fabrics, for twill woven PES fabric with similar thickness and weight as in our study. Thermal resistance was measured by a sweating guarded hot plate. The values of TCC/TCR in this study were closer to the thermal conductivity of polyester fabrics (0.031 W/m °C) measured by an Alambeta instrument as reported by Yavascaoglu et al. [31] for polyester woven fabrics with comparable bulk density but lower than in the study of Özkan et al. [32]. They have also used the Alambeta instrument to investigate the thermal conductivity of 11 polyester knitted fabrics differentiated by yarn type and knit structure and reported values between 0.042–0.048 W/m°K. Alambeta measures thermal resistance, conductivity and absorbtivity of the fabrics kept between the hot and cold plates at a defined pressure and as soon as the hot plate touches the fabric surface, the amount of heat flow from the hot surface to the cold surface through the fabric is detected by heat flux sensors [32]. In a recent study Wei et al. [21] used a laser flash apparatus (LFA) that can determine the thermal diffusivity and specific heat capacity to calculate the thermal conductivity. They have investigated the effect of a graphene screen printed on the whole surface of a cotton fabric for welding protective clothing, having a higher weight and bulk density than those in our study. They have reported significant anisotropy values, showing that the fabric has higher in-plane thermal conductivity (2.082 W/m°K) as compared with cross-plane thermal conductivity (0.48 W/m°K).

None of the above studies have assessed the thermal conductivity by the same FTT equipment, the substrate and the application method of graphene were not identical as in our case. It is therefore not fair to compare TCR/TRR values with the thermal conductivity values from other studies. Nevertheless, the values of the thermal conductivity measured by the FTT are in fair agreement with values obtained via other equipment, for comparable polyester fabrics without a graphene treatment. The unexpected low values of the thermal conductivity suggest that FTT is not recommended for conductive fabrics with graphene printed patterns. The FTT is measuring the heat flux passing transversely through the fabric during about 120 s measuring time. Thermal imaging showed that once in contact with a warm surface, the fabric instantly dissipates heat on its surface, within a few seconds along the graphene lines. This is shown and discussed in next Section 3.3.2.

#### 3.3.2. IR Thermography

**Figure 4:**
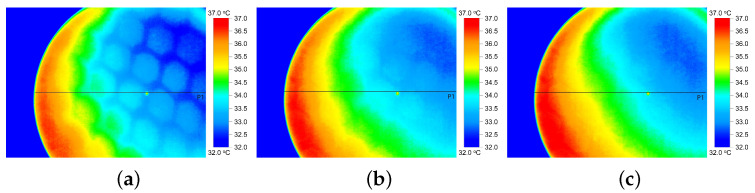
IR thermal images of fabric HC-pattern after 10 s (**a**), 20 s (**b**) and 30 s (**c**) exposure to heat.

**Figure 5:**
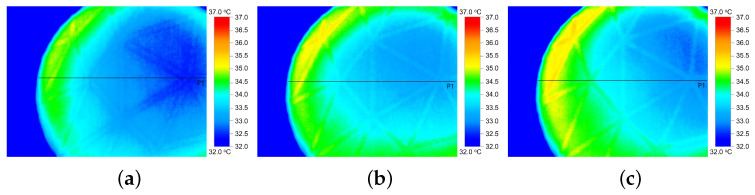
IR thermal images of fabric SW-pattern after 10 s (**a**), 20 s (**b**), and 30 s (**c**) of exposure to heat.

In addition to the thermal conductivity indexes (TCC and TCR) calculated by FTT, the thermal properties of the fabrics were investigated by IR-imaging. The IR-thermograms in Figure 4 and Figure 5 display the temperature distribution on the surface of the HC and SW fabrics, respectively, after 10 s (a), 20 s (b) and 30 s (c) exposure to heat. The temperature of the fabrics varies roughly between 32 °C (blue color) and 37 °C (red color).

**Figure 6:**
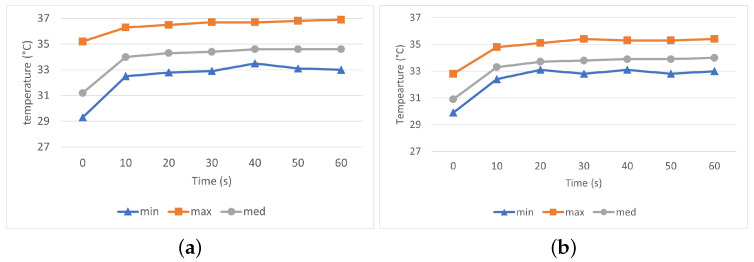
Surface temperature profile of fabric HC (**a**) and SW (**b**) at given measurement moments.

Temperature profile (minimum, maximum, and median) along a horizontal line crossing the IR thermogram (Figure 4 and Figure 5) was computed and is shown in Figure 6 for fabric with HC pattern (a) and SW pattern (b), respectively. In general, it can be seen that the maximum temperature reached on the surface of SW fabric was slightly lower as compared with the HC fabric.

**Figure 7:**
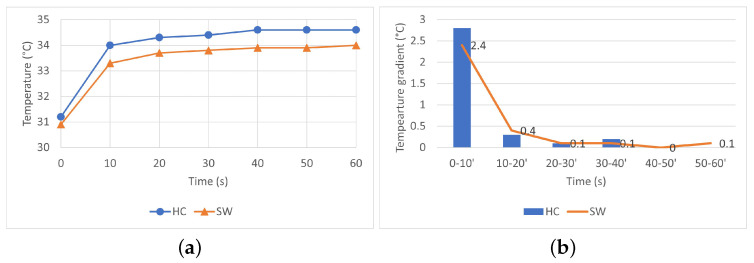
Surface temperature profile (**a**) and gradient (**b**) of fabric HC and SW.

Analysis of thermograms showed a higher temperature of the HC fabric surface after 1 min of heat exposure, as shown in Figure 7a. The temperature of fabric HC increased steeper with about 2.5 °C in the first 10 s and additional 0.4 °C after another 10 s following the fabrics exposure to heat, Figure 7b. The results show that fabric HC has a better heat dissipation and faster response than fabric SW. The results suggest that tight-fitted sportswear with HC-pattern have greater potential to rapidly spread the heat from hot skin areas and would, therefore, offer a better sensation of cooling than fabrics SW.

Similar sharp increase in surface temperature within the first 10 s of 3–5 °C followed by relative constant temperature was also reported in [13] for fully treated graphene cotton fabrics. Our results are also in agreement with the ones presented in [4] where has been concluded that the graphene coating provided a better thermal dissipation, with a maximum difference in the surface temperature between the coated fabric and control of 2.6 °C. When compared with the FTT, the thermal properties of the two fabrics and the differences thereof seem to be better described by IR-thermography that clearly shows quick heat distribution along the two graphene patterns.

### 3.4. Fabric Thermophysiological and Sensorial Comfort Properties

#### 3.4.1. Thermophysiological Comfort Properties 

Mean values (±SDEV) of the air permeability, water vapor permeability and moisture drying time of the two fabrics are displayed in Table 7.

Fabric air permeability together with clothing design directly affects clothing ventilation. Dry and evaporative heat loss are highly affected by air exchange between the clothing micro-environment and the external environment. Increase in the air exchange through the fabrics and the apertures of the garment reduces the thermal insulation and the evaporative resistance [33]. The fabric with the SW pattern exhibits a higher air permeability (29.8 mm/s) than fabric HC (18.6 mm/s). Both fabric HC and SW showed a significant increase in the air permeability upon 10% and 20% biaxial stretch. For fabric HC the air permeability increases with 144% (BS 10) and 1178% (BS20) and for fabric SW with 363% (BS10) and 938% (BS 20), respectively. These values are in line with other studies [29] that reported a significant increase in air permeability of a stretch fabric upon bilateral stretch of 10%. The denser HC-graphene pattern seems to lead to lower air permeability when compared to the SW-fabric. The differences were statistically significant for fabric in relaxed state RS (*p* = 0.000), as well as in bilateral stretched 10 BS (*p* = 0.004) and 20 BS (*p* = 0.001). Fabric HC has higher bulk density (382.4 g·m${}^{-3}$) as compared with fabric SW (378 g·m${}^{-3}$) and has a denser graphene pattern that covers a larger fabric surface and, therefore, causes a lower air permeability.

Fabrics with elevated water vapor permeability are preferred for sportswear as they allow quick transport of the sweat in vapor state from the skin to the environment and favorize body evaporative cooling. The two graphene patterns lead to some differences in water vapor permeability of the HC and SW fabrics but the differences were not statistically significant (*p* = 0.7). Clothing may become wet or damp during wear because of non-evaporated sweat or rain. The presence of liquid in a fabric increases the effective garment mass resulting in potential discomfort to the wearer and a cold, clammy feeling [34]. The time required for a fabric to dry is therefore important in maintaining a desired level of comfort. The results showed that fabric HC needs significantly more time to dry in the given conditions regardless of the type of artificial perspiration ACP (*p* = 0.03) or ALP (*p* = 0.01) used.

Moisture management properties of the fabrics are summarized in Table 8. Most of the indexes including OMMC are comparable for both fabrics. For instance, both fabrics have reached grade 5 (excellent) wetting time, which is the time in which the top and bottom of the fabrics starts to be wetted.

Absorption rate reflects the ability of the top and bottom surfaces of the fabric to absorb moisture. The fabrics had similar behavior and reached both grade 3 (good) for the top side with the graphene patterns and grade 4 (very good) for the bottom side. Both fabrics reached grade 5 for maximum wetted radius and spreading speed, which reflects the moisture spreading ability and the spreading speed to reach the max wetted radius, respectively. The spreading speed is an indication of a fabric’s ability to dry quickly. Fabric SW has a higher spreading speed than fabric HC, in agreement with the drying time (Table 7).

Accumulative one-way transport capacity (R) is the difference in the cumulative moisture content between the top and bottom surfaces of the fabric and a direct indication of a fabric’s ability to allow liquid that wets the inner surface to move towards the outer surface. The R-index is particularly linked to the comfort, as any trapped moisture in the inner surface over a long period will result a great physiological discomfort to the wearer. Fabric HC had a fair capability to transport moisture in contrast to fabric SW that showed a good accumulative one-way transport index.

The overall (liquid) moisture management capability (OMMC) reflects the overall capability of a fabric to transport liquid moisture and is calculated by combining three measured attributes of performance: the liquid moisture bottom absorption rate, the accumulative one-way liquid transport capability (R), and the bottom moisture spreading speed. Both fabrics exhibited comparable and good (grade 3) OMMC values (0.6). Fabric SW was classified as water management fabric and seems to perform better than fabric HC, which has a lower R-index and was classified as fast absorbing quick drying fabric.

#### 3.4.2. Fabric Sensorial Properties

The primary sensorial comfort indexes, such as smoothness, softness, warmness, total hand, and total feel predicted by the FTT device are listed in Table 9. Despite a more dense graphene pattern, fabric HC outperforms fabric SW with respect to the primary sensory smoothness, softness, and coolness, both at active (hand) and passive (touch) evaluation. Passive evaluation means wearing the fabric on the skin and active evaluation means assessment of the fabric with the hand. The total hand and total touch are calculated by the FTT based on the three primary comfort indices corresponding to active and passive evaluation, respectively.

Lower values (0.36/0.46 vs. 0.32/0.38) of the warmness predicted by the FTT (Table 9) suggest that the outside of the fabric HC (with graphene pattern) feels cooler than fabric SW both by active assessment (hand) and passive assessment (touch). Nevertheless, the FTT classified fabric HC as cooler (warmness grading 2) than fabric SW (warmness grading 3) only for active assessment (hand), as shown in Table 10. This is in agreement with results of IR camera (Figure 4 and Figure 5) that generally show a faster increase in the HC fabric surface temperature, and has, thus, a greater potential for a better cooling sensation of the wearer.

## 4. Conclusions

The results show that fabric SW performs better than fabric HC with respect to air permeability, drying time, moisture, and liquid management properties. This fabric is likely to enable evaporative cooling of the body and has great potential in sport activities with elevated sweat rate. Fabric HC exhibited better sensorial properties than fabric SW and the IR thermography clearly showed faster dissipation of heat, which suggests that excess heat will dissipate faster into the environment, when the fabric is in tight contact with hot body parts. It is also expected that fabric HC will be more effective in cases of exercising in environments with elevated air humidity, where the evaporative cooling mechanism is not working. Further research envisages further validation of these results through wear trials by assessing differences in the heat strain of the test persons while wearing sportswear in SW and HC fabrics. Differences between the performance and comfort of the two fabrics will be assessed by monitoring physiological parameters (such as core and skin temperature, heart rate) of the test subjects as well as their thermal sensation and comfort.

## Figures and Tables

**Table 1:** Structural and physical properties of the PES-EL plain woven fabrics HC and SW (mean ± SDEV).

Fabric ID	Fabric Composition PES-EL	Graphene Content	Mass per Unit Area	Thickness	Bulk Density	Elongation	
	**%**	**%·m** ${}^{-2}$	**g·m** ${}^{-2.5}$	**mm**	**kg·m** ${}^{-3}$	**Warp**	**Weft**
						**%**	**%**
HC	74/26	30.5	120	0.31	382.4	79	50
			± 3	± 0.01	± 8.1	± 2.4	± 1.4
SW	74/26	23.9	112	0.30	378	66	55
			± 1	± 0.01	± 6.5	± 2.7	± 1.6

**Table 2:** Fabric indices measured by the FTT equipment and their meaning.

FTT	Description	Unit	Usual Interpretation
Index			
BAR${}_{e/a}$	Bending Average Rigidity	gf mm/rad	force needed to bend per radian
BW${}_{e/a}$	Bending Work	gf mm rad	work needed to bend the specimen
SFC${}_{e/a}$	Surface Friction Coefficient	-	friction coefficient on surface with ribbed metal plate
SRA${}_{e/a}$	Surface Roughness Amplitude	µm	amplitude of irregular wavelength
SRW${}_{e/a}$	Surface Roughness Wavelength	mm	wavelength of irregular wavelength
CW	Compression Work	gf mm	work needed to compress the specimen
CRR	Compression Recovery Rate	-	percentage of thickness changes after compressed
CAR	Compression Average Rigidity	gf/cm${}^{2}$ mm	force needed to compress per mm
RAR	Recovery Average Rigidity	gf/cm${}^{2}$ mm	force reflected when recovery per mm
TCC/TCR	Thermal Conductivity during Compression/ Recovery	10${}^{-3}$ W/m °C	energy transmitted per degree per mm when the specimen during compression/recovery period
Qmax	Thermal maximum flux	W/m${}^{2}$	maximum energy transmitted during compression

**Table 3:** Mean values (±STDEV) of various mechanical and thermal properties of the fabrics with honeycomb (HC) and spiderweb (SW) graphene pattern as measured by FTT equipment for the fabric inside and outside, weft wise, and warp wise.

Fabric Index	Honeycomb (HC)		Spiderweb (SW)		Honeycomb (HC)		Spiderweb (SW)	
	Fabric Outside				Fabric Inside			
	Weft (e)	Warp (a)	Weft (e)	Warp (a)	Weft (e)	Warp (a)	Weft (e)	Warp (a)
BAR${}_{e}$ / BAR${}_{a}$	45.66	47.59	44.46	36.51	35.73	42.99	36.69	25.58
(gf mm/rad)	± 12.74	± 7.23	± 3.88	± 6.29	± 14.31	± 6.73	± 5.35	± 12.83
BW${}_{e}$ / BW${}_{a}$	222.22	211.76	162.7	148.84	233.31	160.51	183.73	124.83
(gf mm rad)	± 46.43	± 8.99	± 18.61	± 13.27	± 11.24	± 52.7	± 22.73	± 52.29
SFC${}_{e}$ / SFC${}_{a}$	0.36	0.23	0.31	0.30	0.23	0.34	0.21	0.36
(-)	± 0.08	± 0.01	± 0.02	± 0.015	± 0.01	± 0.01	± 0.02	± 0.09
SRA${}_{e}$ / SRA${}_{a}$	22.31	46.16	22.25	45.15	35.58	44.63	26.19	42.19
(μm)	± 9.79	± 27.53	± 9.56	± 26.53	± 16.68	± 22.34	± 15.07	± 19.8
SRW${}_{e}$ / SRW${}_{a}$	2.36	4.39	3.15	3.6	3.77	4.65	2.9	3.97
(mm)	± 1.23	± 3.32	± 2.26	± 1.83	± 2.62	± 2.8	± 2.06	± 2.33
CW (gf mm)	108.02 ± 17.32		235.96 ± 271.41		111.58 ± 22.57		182.84 ± 172.61	
CRR (-)	0.42 ± 0.04		0.37 ± 0.06		0.42 ± 0.02		0.38 ± 0.05	
CAR (gf/cm${}^{2}$ mm)	2354.5 ± 465.78		1781.15 ± 909.03		2449.4 ± 692.9		1970.37 ± 869.34	
RAR (gf/cm${}^{2}$ mm)	4582.73 ± 896.71		3968.05 ± 1679.78		4526 ± 878.31		4433.3 ± 1783.26	
TCC (10${}^{-3}$ W/m ${}^{\circ }$C)	33.63 ± 1.3		34.94 ± 6.63		34.12 ± 0.85		32.35 ± 1.44	
TCR (10${}^{-3}$ W/m ${}^{\circ }$C)	32.9 ± 0.88		34.12 ± 6.12		33.46 ± 0.54		31.41 ± 1.31	
Qmax (W/m${}^{2}$)	1162.67 ± 10.87		1154.65 ± 61.66		1163.07 ± 6.61		1150.39 ± 58.41	

**Table 4:** T-test results for the fabric outside, showing significant differences (*p* < 0.05) between FTT fabric indexes depending on fabric (HC and SW) or fabric direction (weft-warp), in the case of bending, friction, and roughness properties.

Fabric Outside																	
Fabric indexes	BAR${}_{a}$	BAR${}_{e}$	BW${}_{a}$	BW${}_{e}$	CW	CRR	CAR	RAR	TCC	TCR	Qmax	SFC${}_{a}$	SFC${}_{e}$	SRA${}_{a}$	SRA${}_{e}$	SRW${}_{a}$	SRW${}_{e}$
Fabric SW versus fabric HC																	
*p*-values	0.09	0.80	**0.002**	0.06	0.33	0.19	0.25	0.49	0.66	0.68	0.79	0.38	0.20	0.94	0.96	0.62	0.54
Weft versus warp																	
HC	0.82		0.64		-	-	-	-	-	-	-	**0.01**		0.15		0.3	
SW	**0.04**		0.08		-	-	-	-	-	-	-	0.08		0.15		0.77	

**Table 5:** FTT-grading (1—very low, 2—low, 3—middle, 4—high, 5—very high) of HC and SW fabric indexes.

Fabric Index	Honeycomb (HC)		Spiderweb (SW)		Honeycomb (HC)		Spiderweb (SW)	
	Fabric Outside				Fabric Inside			
	Weft	Warp	Weft	Warp	Weft	Warp	Weft	Warp
BAR	1	1	1	1	1	1	1	1
BW	1	1	1	1	2	2	1	1
SFC	2	2	2	2	2	3	1	2
SRA	1	1	1	1	1	2	1	1
SRW	1	1	1	1	1	2	1	1
CW	1		1		1		1	
CRR	2		2		2		2	
CAR	3		3		3		3	
RAR	2		1		1		2	
TCC	2		2		2		2	
TCR	2		2		2		2	
Qmax	3		3		3		3	

**Table 6:** T-test results for the fabric inside, showing significant differences (*p* < 0.05) between FTT fabric indexes depending on fabric (HC and SW) or fabric direction (weft-warp).

Fabric Inside																	
Fabric indexes	BAR${}_{a}$	BAR${}_{e}$	BW${}_{a}$	BW${}_{e}$	CW	CRR	CAR	RAR	TCC	TCR	Qmax	SFC${}_{a}$	SFC${}_{e}$	SRA${}_{a}$	SRA${}_{e}$	SRW${}_{a}$	SRW${}_{e}$
Fabric SW versus fabric HC																	
*p*-values	0.13	0.09	0.21	**0.008**	0.40	0.11	0.28	0.90	**0.02**	**0.008**	0.63	0.59	0.06	0.88	**0.002**	0.75	0.10
Weft versus warp																	
HC	0.34		**0.03**		-	-	-	-	-	-	-	**0.000**		0.60		0.72	
SW	0.06		0.06		-	-	-	-	-	-	-	**0.02**		0.21		0.48	

**Table 7:** Air permeability of the HC and SW fabrics (mean ± SDEV) in relaxed state (RS) and under 10% and 20% biaxial stretch (10 BS and 20 BS), water vapor permeability and drying time of the fabrics wetted with acid (ACP) or alkaline (ALP) perspiration (ISO 105-E04: 2013).

		SI Unit		Honeycomb (HC)	Spiderweb (SW)
Air permeability		mm/s	RS	18.6 ± 4	29.8 ± 4
			10 BS	97.5 ± 11	138 ± 9
			20 BS	237.8 ± 8	309.2 ± 15
Water vapor permeability		g/m${}^{2}$.Pa.h	-	0.21 ± 0.04	0.19 ± 0.04
Drying time		min	ACP	33 ± 0.4	26 ± 0.9
			ALP	34 ± 0.4	28 ± 1.4

**Table 8:** Moisture management properties of the fabrics (mean ± SDEV) with graphene pattern honeycomb (HC) and spiderweb (SB).

		Unit	Honeycomb (HC)	Spiderweb (SW)
Wetting Time Top		s	2 ± 0.4	2.1 ± 0.2
Wetting Time Bottom		s	2.8 ± 0.1	2.6 ± 0.3
Top Absorption Rate		%/s	62 ± 3.9	57.7 ± 11.8
Bottom Absorption Rate		%/s	69 ± 5.4	74.3 ± 17.3
Top Max Wetted Radius		mm	28 ± 2.6	27.2 ± 3.6
Bottom Wetted Radius		mm	30 ± 0.0	28.9 ± 2.2
Top Spreading Speed		mm/s	6.8 ± 0.8	7.5 ± 1.6
Bottom Spreading Speed		mm/s	6.4 ± 0.9	7 ± 1.2
Accumulative one-way transport index (R)		%	116.7 ± 15.8	131.8 ± 49.9
OMMC			0.6 ± 0.0	0.62 ± 0.0

**Table 9:** FTT-predicted primary and global comfort indexes (mean ± SDEV) of the HC and SW fabrics.

		Honeycomb (HC)	Spiderweb (SW)
Smoothness	Hand	0.72 ± 0.09	0.65 ± 0.12
	Touch	0.88 ± 0.08	0.81 ± 0.12
Softness	Hand	0.72 ± 0.05	0.73 ± 0.04
	Touch	0.71 ± 0.04	0.68 ± 0.07
Warmness	Hand	0.36 ± 0.07	0.46 ± 0.15
	Touch	0.32 ± 0.04	0.38 ± 0.08
Total hand of the fabric		0.63 ± 0.04	0.62 ± 0.04
Total touch of the fabric		0.73 ± 0.05	0.69 ± 0.07

**Table 10:** FTT-grading (1—very low, 2—low, 3—middle, 4—high, 5—very high) of HC and SW fabric.

		Honeycomb (HC)	Spiderweb (SW)
Smoothness	Hand	4	4
	Touch	5	4
Softness	Hand	4	4
	Touch	4	4
Warmness	Hand	2	3
	Touch	2	2
Total hand of the fabric		4	4
Total touch of the fabric		4	4

## Data Availability

Not applicable.
